# Geographic range shapes influence species richness in global hotspots

**DOI:** 10.1126/sciadv.aea0138

**Published:** 2025-08-13

**Authors:** Jesper Sonne, Michael Krabbe Borregaard, Robert K. Colwell, Carsten Rahbek

**Affiliations:** ^1^ Center for Global Mountain Biodiversity, Globe Institute, University of Copenhagen, Universitetsparken 15, 2100 Copenhagen, Denmark.; ^2^ Globe Institute, University of Copenhagen, Copenhagen, Denmark.; ^3^ Department of Ecology and Evolutionary Biology, University of Connecticut, Storrs, CT, USA.; ^4^ University of Colorado Museum of Natural History, Boulder, CO, USA.; ^5^ Center for Macroecology, Evolution and Climate, Globe Institute, University of Copenhagen, Universitetsparken 15, 2100 Copenhagen Ø, Denmark.; ^6^ Department of Biology, University of Southern Denmark, 5230 Odense M, Denmark.

## Abstract

The extraordinary richness of species in tropical mountain regions is often attributed to aggregations of small-ranged species, allowing tight spatial packing of their ranges. However, ranges of species in these regions are also distinctly more patchy and elongated than those found in adjacent lowlands. Our global analysis of mainland birds demonstrates that these range shapes augment the spatial variation in species richness. Both the linearity and patchiness of species ranges are most pronounced in aseasonal and topographically complex regions, most notably in the tropical Andes, where these range shapes contribute more to the pattern of species richness than broad-scale variations in range size. Consequently, niche-based processes that govern the spatial configuration of habitats contribute to patterns of species richness in global hotspots over and above the processes that affect species richness through range sizes.

## Teaser

Elongated and patchy bird ranges increase species richness in global biodiversity hotspots beyond the effect of small range sizes.

## INTRODUCTION

**Fig. 1.**
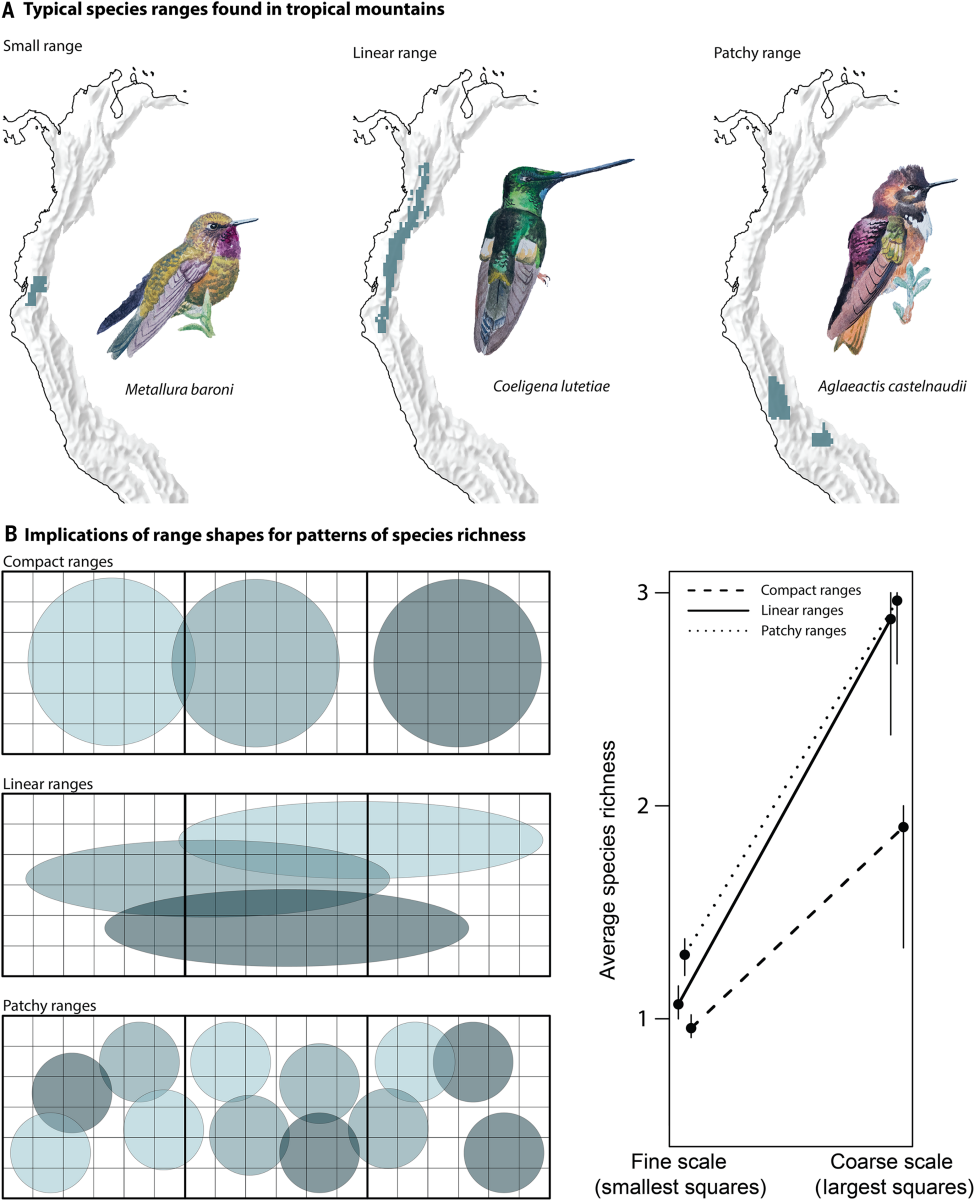
Conceptual depiction of how species range shapes affect spatial patterns of species richness. Global hotspots of species richness in tropical mountain regions are typically characterized by species with small, linear, or patchy distributions. (**A**) Examples of such species from the tropical Andes, where avian species richness reaches its global maximum. As with small range sizes, linear and/or patchy range shapes may amplify the number of species at coarse spatial resolutions. (**B**) This pattern is illustrated for three species (shades of blue) with equal range sizes distributed in a rectangular domain of 6 × 18 small quadrats. Randomly relocating the species ranges on the domain results in distinct patterns of species richness, dependent on the shapes of species ranges. The relocation of ranges was done by randomly selecting a range centroid on the domain. Ellipses and circles were fully contained within the domain, with no parts extending beyond its boundaries. All ellipses had the same horizontal orientation. At the coarse spatial resolution (largest squares), both linear and patchy range shapes generate consistently higher species richness than species with compact, coherent ranges. This emergent pattern is spatially scale-dependent, diminishing toward the finer spatial resolution (smallest squares). Vertical bars on the line plot correspond to the 95% variation of the randomization. Drawings in (A) by J. Fjeldså.

Tropical mountain regions are global hotspots of species richness, characterized by high concentrations of small-ranged species that replace one another along steep elevational gradients (*1*). Apart from their small size, the ranges of mountain species also have unique shapes, typically being more elongated or patchy than those from adjacent lowlands [Fig. 1A; (*2*–*4*)]. These particular range shapes may have a profound influence on emergent patterns of species richness, an effect that is distinct from the effect of species’ range sizes. Nevertheless, the consequences of range shapes for the aggregation of species in tropical mountain regions remain largely ignored.

The size and shape of species ranges result from similar but not identical processes (*5*–*7*). The processes that create range shape are always spatially explicit, while the processes determining range size are influenced by intrinsic and scale-independent factors, such as dispersal limitations and life-history traits (*5*, *7*, *8*). Range shapes are largely determined by the spatial configuration of suitable habitats and climates, which limit range expansion but do not explicitly constrain patterns of range sizes (*6*, *9*, *10*). Although the processes are distinct, it follows from classical niche theory that both small range sizes and elongated range shapes may result from selection for narrow environmental climate niches (*11*), which cause turnover in species composition among communities in close geographic proximity (*12*, *13*).

The relative contributions of range sizes and range shapes to patterns of species richness remain an open question. The biogeographical literature has a long history of investigating the implications of species range size frequency distributions (*14*–*19*), while the patterns of range shape have attracted much less attention (*3*, *6*, *10*, *20*). Nevertheless, the shapes of species’ habitats and realized ranges may have implications for range overlap and large-scale patterns of species richness. For example, in tropical mountain regions, niche conservatism increases the likelihood that populations become isolated within narrow, elongated zones of habitats. If allopatric speciation subsequently occurs, species richness may be elevated in secondary contact zones, where the distributions of related species overlap (*21*). Given that elongated distributions of species are parallelly aligned, this increased range overlap within habitats is accompanied by higher species turnover between habitat zones along the mountain slope (*11*), resulting in increased species richness at coarser spatial resolutions (*16*, *22*). Moreover, species with linear or patchy geographic ranges—defined as distributions subdivided into distinct patches at the scale of analysis—often span greater distances than those with compact, cohesive ranges. This broader spatial extent covered by linear or patchy ranges can lead to increased range overlap in specific areas, as such ranges contribute more distributional records at coarser spatial resolutions than species with compact and spatially cohesive distributions of the same size (Fig. 1B). Consequently, range linearity and patchiness increase the accumulation of species within certain areas, over and above the effect of right-skewed range size frequency distributions (*14*, *16*, *19*, *22*–*24*).

Here, we examine how the global pattern of species richness is influenced by the patchiness and linearity of species range shapes. Moreover, we examine how patterns of range shape complement patterns of range size in affecting global hotspots of species richness. Our dataset comprises the breeding distributions of the world’s 10,772 extant bird species, mapped at 1° and 0.25° resolutions, based on the distribution database of Rahbek *et al*. (*25*), as used in (*22*, *26*, *27*). Processes underlying patterns of range size and shape are not comparable between island and mainland regions because of the islands’ disproportionally stronger geometric constraints (*28*). The climatic and topographical constraints, considered in our analysis, are most relevant on large landmasses, as range shapes on islands will largely reflect the size, shape, and isolation of the island itself. Therefore, we concentrate our analyses on global mainland regions, following Pigot *et al*. (*6*). No single metric fully captures variability in range shape. While patchiness, like range size, is a relatively simple property, linearity represents a more complex, multidimensional property, as reflected by the diverse metrics used in the literature (*6*, *10*, *29*). To address this challenge, we base our metrics on comparing each range to counterfactual, biogeographically informed simulations of range shape and placement, under different scenarios. The simulation approach leverages the classic spreading dye algorithm, following Jetz and Rahbek (*30*). Each simulation maintains the size and approximate location of the empirical range while varying two assumptions about range shape (fig. S1). This analytical design does not aim to provide a first-principle explanation of the observed pattern of species richness. Instead, it compares simulated patterns of species richness under different assumptions about range shapes, without depending on any particular metric.

## RESULTS AND DISCUSSION

### Range shapes augment geographic patterns of species richness

**Fig. 2.**
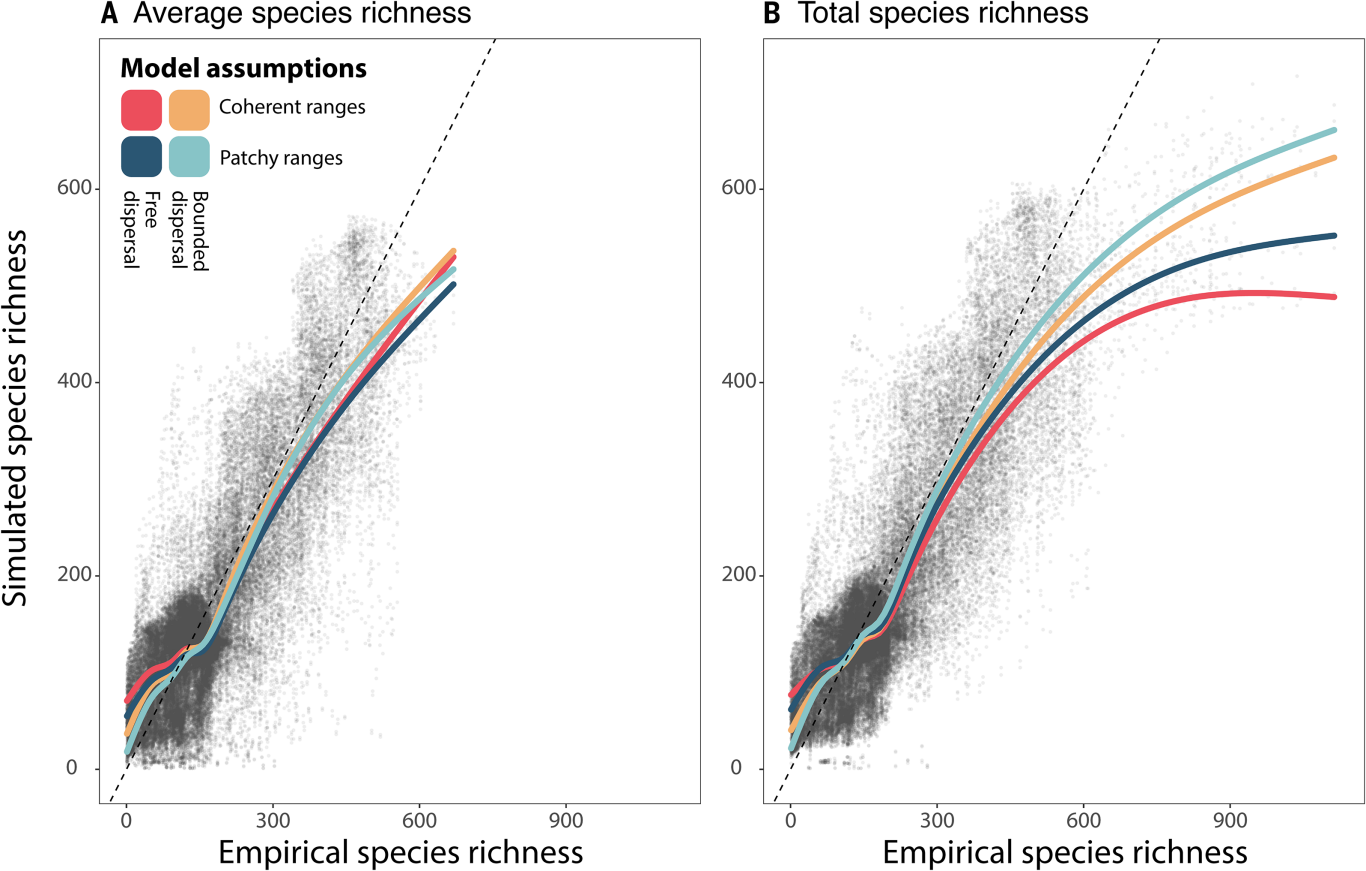
Implications of range shapes for global patterns of species richness from four simulation models in relation to empirical patterns. The scatterplots show the relationship between simulated and empirical species richness at a spatial resolution (i.e., 1° scale resolution). The colored lines correspond to spline regressions for models with different assumptions about range linearity and patchiness. Bounded dispersal within the species’ elevational range limits tends to create linear ranges, while using multiple geographic starting positions creates patchy ranges. The gray points correspond to individual grid cells for each of the four models. The dashed lines have zero intercept and unit slope. (**A**) Species richness averaged at the 0.25° scale resolution. (**B**) Total number of species within each 1° grid cell. Together, the two panels demonstrate that linear and patchy range shapes contribute to the geographic accumulation of species richness, particularly in the world’s most species-rich regions.

We find that species richness at 1° resolution across Earth’s mainlands is amplified by the presence of linear and/or patchy range shapes (Fig. 2). Our simulations underestimates species richness in the most species-rich areas, implying that empirical range boundaries are spatially more constrained than anticipated from the elevational range limits and contemporary climate volume occupied by species, as implemented in our simulations (fig. S2). While our simulation models preserve the empirical range size distribution and the spatial arrangement of ranges, relaxing assumptions that constrain range shape leads to marked underestimation of species richness. By imposing more constraints on range shape, our simulations gradually converge toward the empirical pattern of species richness (fig. S3), suggesting that the effects of range shape on species richness are fundamentally larger than what is detected by comparing our four simulation models.

The effect of range shapes on species richness is expected from a geometric edge effect. For ranges of equivalent sizes, greater linearity and patchiness increase the shape’s perimeter-to-area ratio (*10*, *24*). The enlarged perimeter expands the contact zones between species ranges, increasing range overlap at regional spatial resolutions. We simulated linear range shapes in models that constrain ranges to areas within the elevational range limits and the contemporary climate volume of each modeled species. These spatially explicit constraints on range expansion assume that species have bounded, sometimes linear, dispersal domains that follow the geographic distribution of suitable habitat.

We then simulate range patchiness by specifying multiple starting positions, one for each range patch in the empirical distribution of each species. The inclusion of both range linearity and range patchiness increases the geographic extent covered by the ranges of each species, for a given total range area, which tends to bring local populations in contact with species assemblages on distant elevational transects (see fig. S1 for depictions of each model). In contrast, relaxing these two constraints produces ranges that are more compact and spatially cohesive (i.e., via a von Neumann neighborhood), bounded only by the hard coastal boundaries of the mainland. The greater geographic distance covered by linear and/or patchy ranges does not affect the richness at the 0.25° resolution where the simulations are conducted (Fig. 2A) but drastically increases range overlap at the 1° resolution (Fig. 2B). The contrast between results at the two spatial scales illustrates that range shapes affect patterns of species richness by increasing spatial turnover in species composition. The two-way experimental design, which separates the effects of range linearity and patchiness (fig. S1), confirms an additive effect of these two shape characteristics (Fig. 2B). Together, range linearity and patchiness enhance the apparent accumulation of species richness, particularly in the most species-rich regions of the world, where ranges are often linear and/or patchy.

These results reveal a fundamental factor contributing to the geographic accumulation of species within global hotspots of species richness, complementary to processes causing high concentrations of small-ranged species. This observation is related to findings from the classic conservation literature that elongated nature reserves are likely to include more species than compact reserves of equivalent area (*31*). Our study simply changes the focus to the range shapes of individual species and their implications for species richness within grid cells. Kunin (*31*), working with reserve design, showed that the mechanism holds across varying spatial resolutions (9 to 2500 km^2^), except at the smallest resolutions (≤1 km^2^). This result suggests that the implication of range shapes for the accumulation of species richness is also largely invariant to the spatial scale of resolution.

Our results clearly demonstrate the existence of species richness hotspots at the 0.25° resolution, despite the absence of detectable range shape effects at this spatial scale (Fig. 2). According to our theoretical framework (Fig. 1), the impact of range shapes is inherently undetectable at the resolution at which simulations are executed. Consequently, although range shape likely influences species richness patterns at 0.25° resolution, detecting the effect at such fine spatial scales would necessitate species occurrence data at even finer resolutions. Previous studies investigating the determinants of species richness were mostly conducted at a 1° resolution (*16*, *17*, *26*, *32*, *33*), as were previous macroecological analyses of range shape (*6*, *29*). This coarser resolution captures both biogeographical processes (e.g., speciation-extinction dynamics), landscape/habitat features [but see (*34*)], and, perhaps to some extent, species interactions (*35*). Given these characteristics, along with the extensive use of 1° resolution in macroecological studies of species richness, we considered this scale to be appropriate for our analyses. To demonstrate the implications of range shape at the 1° resolution, we ran the simulation at 0.25° resolution, which we considered the finest resolution that balanced omission and commission errors in the species range data.

The mechanism by which species richness changes according to the shape of nature reserves has traditionally been ascribed to the spatial autocorrelation of environmental conditions and species distributions (*31*). In contrast, our study concerns the geophysical and niche-based processes that determine species range shapes (*6*). These shapes may originate from interactions between niche conservatism (*36*–*38*), geometric constraints (*15*, *30*), and biotic interactions (*39*), ultimately driven by the spatial configuration of suitable habitat (*3*, *40*), complementary to intrinsic characteristics of the species affecting range size frequency distributions [e.g., dispersal limitation; (*5*)]. According to the concept of tropical niche conservatism, patterns of species richness among related species arise from the overlap among geographically structured ranges that follow the spatial configuration of habitats and climatic conditions (*33*, *36*–*38*). Previous studies supported this idea by showing that boundaries of assemblage dispersion fields of South American birds follow the configuration of major vegetation types (*19*, *38*). At a global scale, another study found that the shape of avian ranges did not deviate from a null expectation within tropical latitudes, meaning that ranges of species are either relatively compact or shaped by the geometric constraints of the continental landmass (*29*). The interpretation of this result was that the range shapes of tropical species are mainly determined by stochastic processes (e.g., dispersal limitation). In tropical latitudes, the widespread occurrence of compact ranges may originate from the expansive lowland areas where species exhibit compact distributions. Thus, broad latitudinal patterns of range shape, such as those described by Castro-Insua *et al*. (*29*), are not applicable to ranges found in species-rich tropical mountain regions (*3*, *6*, *10*). For those areas, we show that high species richness can instead result from a strong representation of linear or patchy ranges, providing an explicit explanation for how this niche-based mechanism influences patterns of species richness in conjunction with topographic complexity.

This explanation extends Janzen’s classic hypothesis on niche theory in tropical mountain regions (*11*). The interplay between high topographic relief and the climate regime means that environmental gradients become highly compressed along tropical elevational transects, explaining their right-skewed frequency distribution of range sizes (*1*, *19*, *22*, *41*, *42*). If the region’s topography has an elongated or sky-island-like structure, the pattern of species richness may also increase due to range overlap between species with linear and/or patchy ranges. In this way, the processes determining range shapes contribute to global hotspots of species richness over and above the classic explanations for the accumulation of species richness in regions with high topographic complexity (*11**,* *12*).

**Fig. 3.**
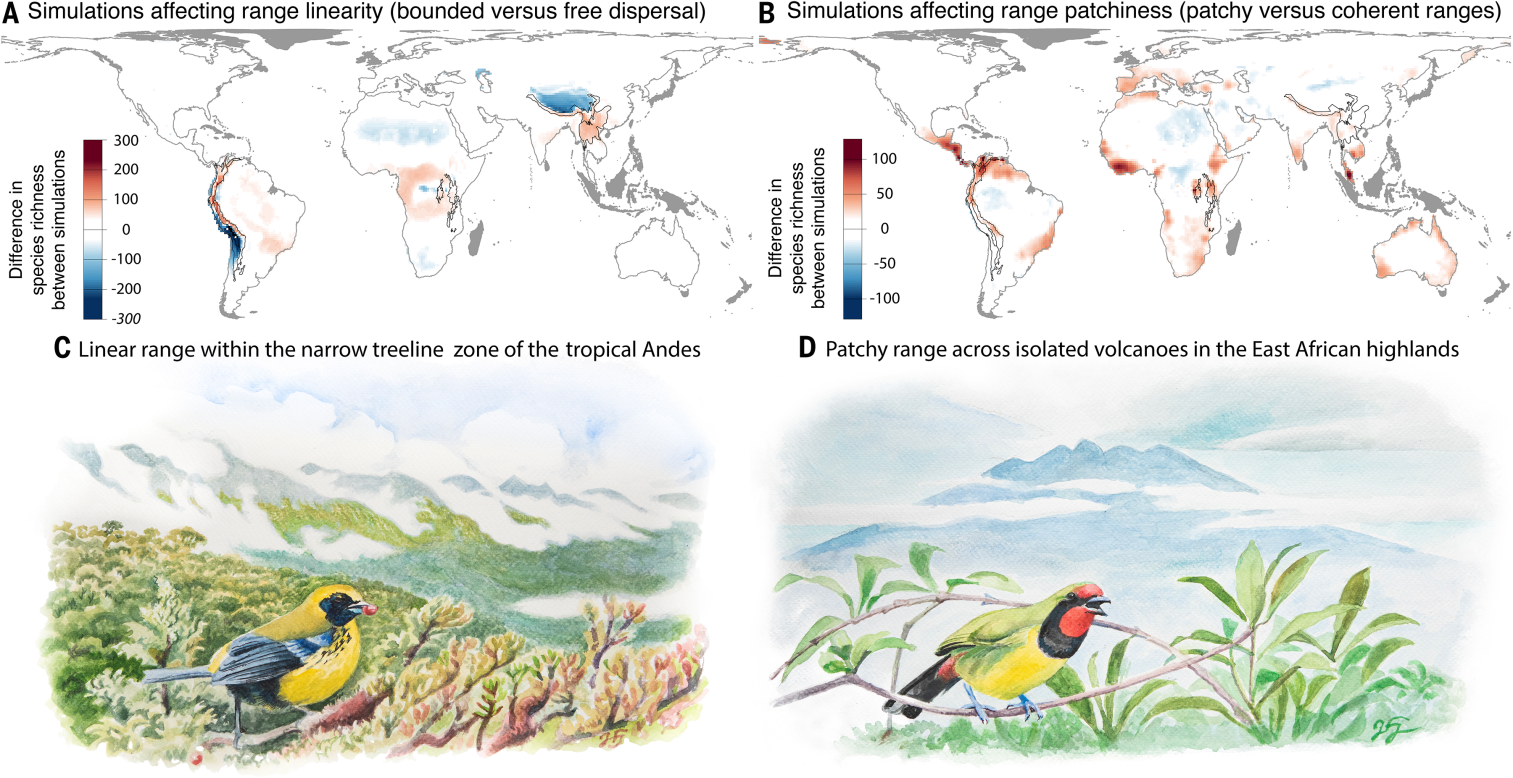
Decomposing the effects of range linearity and patchiness. (**A**) Subtracting models with free dispersal from models with bounded dispersal highlights areas where topographic constraints on range linearity affect species richness. (**B**) Similarly, subtracting models with coherent ranges from models with patchy ranges highlights areas where species richness is affected by range patchiness. Red colors imply elevated species richness as a consequence of range linearity (A) and patchiness (B), whereas blue colors imply reduced species richness. The two maps represent the average two-way comparison between the four models from Fig. 2 (described in fig. S1). See fig. S4 for a comparison between the individual models. The dark gray shades highlight insular regions that were excluded from the analyses. Outlined polygons highlight the most species-rich mountain regions within each continent, following Sonne and Rahbek (*22*). Illustrations, by J. Fjeldså, show examples of species in these regions, with range shapes that match the linear or patchy shapes of their habitats. (**C**) *Tephrophilus wetmorei* has an elongated range along the narrow treeline zone in the Tropical Andes. (**D**) *Telophorus dohertyi* represents a species with a patchy range, reflecting the island-like distribution of montane habitats across isolated volcanoes in the East African highlands.

The impacts of range shape appear to be concentrated in specific areas with the most extreme hotspots of species richness, notably the tropical Andes (Figs. 2 and 3). This region comprises the highest vertebrate species richness globally and has long presented an enigma for understanding the processes that create hotspots of species richness (*1*). Thus, the high spatial concentrations of linear and/or patchy range shapes contribute to explaining the disproportionately high species richness of the tropical Andes compared to other mountain regions (*1*, *22*, *43*). It is the only region where range linearity and patchiness both contribute more to higher species richness than anticipated from the right-skewed range size frequency distribution (Fig. 3). Figure 3 also highlights areas in the Himalayas and the South American Altiplano where range linearity is associated with reduced species richness. This pattern arises because relaxing the assumption of bounded dispersal causes linear-ranged species to expand into adjacent areas along the elevational gradient, resulting in more compact distributions. As a result, richness declines in species-poor regions adjacent to species-rich mountain slopes, where species tend to have elongated distributions. In the Andes, forest birds typically have highly linear distributions, sometimes stretching thousands of latitudinal kilometers (*3*, *4*), in this north-south mountain range. The elevation zones do not represent spatially coherent bands of vegetation, as intersecting dry valleys create dispersal barriers that break up ranges of species into isolated patches (*2*). As a result, individual range patches of many mobile species occur at the same elevations on distinct elevational transects, augmenting the pattern of species richness at the regional scale. Bird ranges in the mountain regions of Eastern Africa resemble the patchy ranges observed in the Andes but lack species with high range linearity (Fig. 3). Consequently, the combined patterns of range shapes predict lower species richness in the mountain regions of Africa compared to those in South America.

### The role of range shape versus range size

**Fig. 4.**
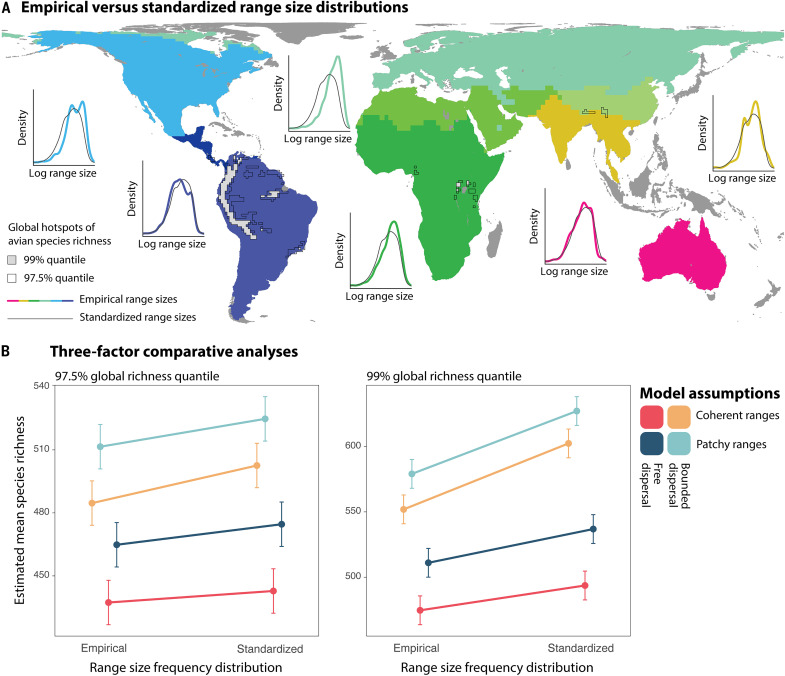
Implications of range shape versus range size for global hotspots of species richness. Comparisons of simulation models with different assumptions about range shape between Earth’s 2.5% and 1% most species-rich terrestrial grid cells at the 1° spatial resolution. Bounded dispersal within the species’ elevational range limits tends to create linear ranges, while initiating the models from multiple geographic starting positions creates patchy ranges. (**A**) Each simulation model was also run with standardized range size frequency distributions, in which the range sizes within each geographic realm (different colors on the map) were replaced by random samples from the global range size frequency distribution. Density plots show the empirical (colored lines) versus standardized (black lines) range size frequency distribution of a focal region. The biogeographical regionalization is based on Holt *et al.* (*26*). The dark gray shading indicates the insular regions excluded from the analyses. (**B**) Linear mixed-effect regressions estimate the mean simulated species richness in models with empirical versus standardized range size, bounded versus free dispersal, and coherent versus patchy ranges. Thus, the analysis resembles a three-factor repeated measures design. Error bars mark the 95% confidence interval estimated by parametric bootstrapping. The results demonstrate that range shapes, overall, contribute more to the accumulation of species within global hotspots of species richness than broad-scale variations in range size.

Global hotspots of species richness, traditionally explained by aggregations of small-ranged species, coincide with areas where linear and/or patchy-ranged species cause the greatest increase in range overlap [Fig. 3; (*1*)]. Thus, the augmented species richness due to linear and patchy range shapes mirrors previous explanations derived from range size frequency distributions (*1*, *14*, *22*). Range size and range shape are mechanistically linked, making it a challenge to disentangle the effects of size and shape analytically. To illustrate the separate effects of range shape and range size, we repeated each simulation model while standardizing the regional range size frequency distribution to that of the global distribution. By applying this standardization, we determine the role of range shapes when there is no biogeographical variation in the range size frequency distribution. The standardization procedure lets species from each of Earth’s major terrestrial realms [following (*26*)] acquire range sizes randomly sampled, without replacement, from the global distribution (Fig. 4A). The randomly sampled range sizes are assigned to each species sequentially, without replacement, to mimic and compare with the empirical rank–range size distribution.

Within the empirical hotspots of species richness (i.e., the 97.5% and 99% global richness quantiles), our standardization that controlled for broad-scale difference in range size had a smaller effect on the simulated pattern of species richness compared to the varying assumptions of range shapes (Fig. 4B). This result holds within the tropical Andean region (which comprises most of the 99% global richness quantile)— renowned for its extraordinary concentration of small-ranged species (*1*). We acknowledge that our analysis does not capture regional-scale patterns of species range sizes, such as the differences between adjacent lowland and highland regions. Nevertheless, broad-scale patterns of range size frequency distributions remain important determinants of the geographic accumulation of species richness. Storch *et al*. (*18*) showed that continent-specific species-area relationships collapse into a universal curve when standardized by the average range size within each continent. Our results suggest that aggregations of small-ranged species are inadequate to characterize global hotspots of species richness found in tropical mountain regions (*1*, *14*, *22*) at spatial resolutions commonly used to depict and study richness. Instead, the two patterns complement each other: Range restrictedness reduces range overlap along and across the elevational gradient, whereas range linearity and patchiness increase range overlap within distinct zones of habitat along the elevational gradient. Both patterns increase the turnover in species composition over short geographic distances, ultimately resulting in high species richness at the broader biogeographical scale (*22*).

Range size is only one of several fundamental attributes of species ranges that determine global hotspots of species richness. Our findings show that the richest of these hotspots are also profoundly influenced by the shapes of species ranges. The result evokes a mechanism—originating from the conservation literature—concerning the design of larger protected areas, in which species richness increases with the linearity and patchiness of nature reserves. Analogously, we demonstrate that species richness in grid cells can be increased by the linearity and patchiness of species ranges. Patterns of range linearity and patchiness are ultimately a consequence of geophysical processes and the breadth of species niches determining the spatial configuration of species’ habitats. Patterns of heightened range linearity and patchiness are most pronounced in topographically complex regions, notably the tropical Andes—renowned for its extraordinary richness of species at regional to continental scales. Hence, our results nuance the established view that global hotspots of species richness in mountain regions merely derive from the aggregation of small-ranged species. It appears that elongated and/or patchy range shapes increase the geographic accumulation of species richness over and above what would be expected, given the climate regime of biomes and the processes determining range size frequency distributions.

## MATERIALS AND METHODS

### Species distribution data

The species range data consist of breeding distributions for all 10,772 birds in the world following the International Ornithological Community taxonomy v.10.2 (*44*) and are compiled from primary sources at the 1° scale resolution (*25*). In addition, we retrieved independent information on the minimum and maximum elevational range limits of each species from independent primary sources (*45*). We used these data in conjunction with a digital terrain model at a 2–arc min resolution (*46*) to project the ranges of species to maps at the 0.25° resolution. A species was considered present in 0.25° grid cells containing elevations within its elevational range limits and within its range envelope. This operation reduces range overlap between species distributed in tropical mountain regions, decreasing the errors of commission (apparent but false range overlap) at the 1° scale (*47*). Species distributed on islands are outliers in a comparative analysis of range shape. Because of the disproportionately stronger geometric constraints on islands, island species tend to have smaller and more patchy distributions than otherwise equivalent species from mainland regions (*48*). Therefore, we exclude all insular regions from our analyses along with any associated island endemics, resulting in a global mainland dataset consisting of 8317 bird species.

### Biogeographical simulations

We simulated the range of each species using a spreading dye algorithm, following the procedure described by Jetz and Rahbek (*30*). The algorithm begins by sampling a random grid cell from the empirical range. From this starting position, the algorithm stepwise selects an adjacent grid cell, using Von Neumann neighborhood, until the number of sampled grid cells corresponds to the empirical range size of the species (Fig. 2). In a geographic domain, the algorithm is not always able to sample a full range within a single coherent domain patch. In this case, the algorithm continues sampling grid cells beyond the hard boundaries of the domain patch. At the first encounter of an unoccupied grid cell within the geographic domain, the algorithm deletes all previously sampled grid cells falling outside the geographic domain. The entire procedure was repeated 1000 times for each species, after which we calculated the average local species richness and the total species richness at the 1° scale resolution. The biogeographical simulations were conducted in Julia using the SpreadingDye.jl package (*49*).

### Geographic constraints on range shape

The shape of species ranges follows the geographic configuration of the landmass in which they disperse. With no constraints on range shape (i.e., “free dispersal”), the shape of species ranges is limited only by the hard boundaries of the coastlines. We began our simulations with such free dispersal, where the entire global mainland region constitutes the geographic domain. This assumption generates compact range shapes (approximately circular) that are restricted only by the hard boundaries of the coastlines (fig. S1A). We then introduced geographic constraints on range shapes based on (i) the empirical minimum and maximum elevational range limits of each focal species, and (ii) the contemporary climate conditions occupied by the species. To characterize the contemporary climate volume, we calculated the minimum convex polygon that encompasses all combinations of mean annual temperature (bio1) and mean annual precipitation (bio12), as well as temperature seasonality (bio4) and precipitation seasonality (bio15), within the empirical range of each species. The climate data were retrieved from the CHELSA-BIOCLIM+ database version 2.1 at a 30–arc sec resolution (*50*), aggregated to a 0.25° resolution. In this scenario, the geographic domain includes only the grid cells that fall within both the elevational range limits and the occupied climate conditions of a focal species. These constraints resulted in geographically structured, often linear range shapes bound to the geographic configuration of suitable habitat (fig. S1B).

### Assumptions of range coherency versus patchiness

Initially, we simulated species ranges starting from a single grid cell, which generated coherent ranges limited only by the patchiness of the geographic domain. However, species often have patchy and disjunct ranges that may cover greater extents than coherent ranges of equivalent sizes (Fig. 1). To capture this effect, we introduced range patchiness as an additional assumption in the biogeographical simulations by allowing multiple starting positions, one for each of the species’ empirical range patches (fig. S1, C and D). We defined range patches as occupied grid cells connected by the Moore neighborhood.

Often, species have tiny range “patches” that do not represent vicariant distribution patterns but small extensions of the larger coherent range (e.g., the tiny grid cell patches in the range of *Coeligena lutetiae*; Fig. 1A). Therefore, such tiny patches were not given independent starting positions in the simulation models assuming range patchiness. Instead, we used an algorithmic procedure to treat the tiny patches as an extension of the larger coherent range, rather than independent range patches. This procedure uses two parameters: first, the minimum patch size relative to the largest patch (PS_min_), and second, the minimum distance between range patches (*d*_min_). The algorithm selects, stepwise, all range patches smaller than PS_min_ and identifies their nearest neighbor patch. If the distance is less than *d*_min_, the algorithm will consider the two neighbors as an associated patch group. This procedure repeats until there are no remaining patches smaller than PS_min_ located less than *d*_min_ from a neighboring patch. We used a default value of PS_min_ = 10% and *d*_min_ = 5 grid cell distance. We selected these parameter sets to best distinguish true range vicariance from tiny patches protruding from coherent ranges (see examples in fig. S5).

### Range size standardization

We investigated how well the species richness simulated by each simulation model coincides with large-scale empirical patterns of range size frequency distributions. Specifically, we reran each simulation model with range sizes within the mainland realms of the world standardized to match the global range size frequency distribution, as defined by Holt *et al.* (*26*). This standardization allowed us to isolate the effect of range shape versus range size on the emergent patterns of species richness. For the species found in a focal realm, we randomly sampled an equivalent number of ranges from the global pool of range sizes and assigned them to the species in size-rank order to match the empirical rank order within the realm (fig. S6). Thereby, the smallest ranges in a modeled focal realm acquire the smallest ranges from the random sample and vice versa for the largest ranges. For species occurring in multiple realms, we assigned the average sampled range size from each realm. This procedure was repeated 1000 times, and species were assigned the average range size (exemplified by the black lines in Fig. 4A’s density plots) from the 1000 runs. For the simulation models maintaining range patchiness, the range standardization added or subtracted grid cells from the empirical range size, proportional to the sizes of the range patches. In this way, the range standardization did not change the relative size differences between the range patches. Under the assumption of bounded dispersal, standardized range sizes occasionally exceeded the geographic domain. In these instances, simulated ranges were constrained to match the size of the geographic domain.

### Analyses

To compare fine versus coarse-scale patterns of species richness, we mapped the average and total species richness at a scale of 1° resolution. Then, we used spline regressions to visualize the relationship between observed and simulated species richness. The simulation models vary in two assumptions: spatially explicit dispersal limitations versus free dispersal and range patchiness versus range coherency, allowing for a two-way repeated measures design.

We identified global hotspots of avian species richness using both 97.5 and 99% empirical richness quantiles at the 1° scale resolution. For these hotspots, we include an additional set of simulation models based on standardized range sizes. We then applied linear mixed-effect regression to estimate the average difference in predicted species richness among the simulation models, using the “lme4” package in R (*51*). In our analysis, we treated grid cell ID as a random effect, while the empirical versus standardized range size, bounded versus free dispersal, and coherent versus patchy ranges were treated as categorical fixed effects, corresponding to a three-way repeated measures design. We computed the estimated marginal means for each combination of predictor variables using the parametric bootstrap test from the “emmeans” R package (*52*). The denominator degrees of freedom, used for estimating the confidence intervals, were estimated using the Kenward-Roger approximation.

## Supplementary Materials

### This PDF file includes:

Figs. S1 to S6 References
