# Supplementary material for: Geographic range shapes influence species richness in global hotspots
Source: Sci Adv. 2025 Aug 13;11(33):eaea0138. doi: 10.1126/sciadv.aea0138 (PMC12346262; doi:10.1126/sciadv.aea0138)
Supplement: Supplementary file 1 — Figs. S1 to S6 References [file sciadv.aea0138_sm.pdf]

Science Advances
AAAS

Supplementary Materials for

Geographic range shapes influence species richness in global hotspots

Jesper Sonne *et al.*

Corresponding author: Jesper Sonne, jesper.sonne@sund.ku.dk

Sci. Adv. **11**, eaea0138 (2025)
DOI: 10.1126/sciadv.aea0138

This PDF file includes:

Figs. S1 to S6
References

fig. S1: Schematic representation of the four simulation models used to simulate species ranges with different shapes. Each panel shows the result of a single model iteration based on the empirical distribution of the hummingbird, *Phlogophilus harterti,* found in the Peruvian Andes. Each model simulates ranges starting from a single grid cell (the black grid cell) randomly selected from within the species' empirical range (dashed polygons in 0.25-degree resolution). From this starting grid cell, the algorithm stepwise selects a von Neumann neighbouring grid cell until *n* grid cells have been sampled, corresponding to the species' empirical range size. Model 1 has the fewest constraints, as the species can disperse anywhere on the map (grey zone) from the starting position. This simulation model tends to generate compact range shapes that are restricted only by the geometric constraints of the continental coastlines. In contrast, Model 2 allows species to disperse only within their minimum and maximum elevational range limits, following Stotz et al. 1996 (53). This constraint generates range shapes that are bound to the spatial configuration of the topographic landscape. The resulting ranges become geographically structured (usually linear, in mountain cordilleras), following the elevational zones of habitats. Models 3 and 4 are similar to 1 and 2 but relax the assumption of range coherency, allowing multiple starting positions, one for each disjunct empirical range patch. Thus, the four simulation models resemble a two-factor repeated-measures analysis. Comparisons of Models 1 vs. 2 and 3 vs. 4 assess the effect of topographic dispersal constraints on species range shape (solid arrows). By contrast, comparing Models 1 vs. 3 and 2 vs. 4 reveals the effect of range patchiness (dashed arrows).

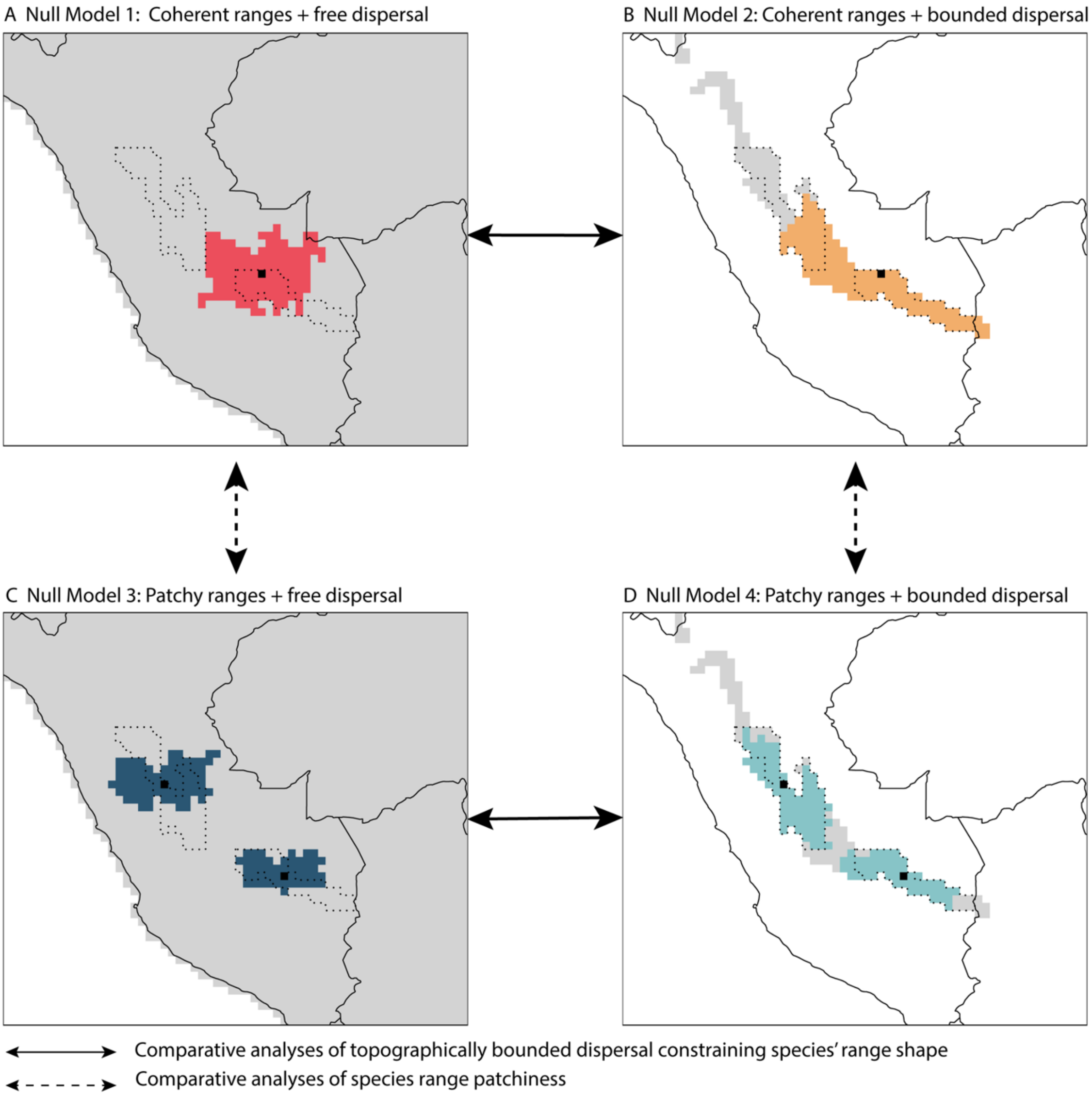
A Null Model 1: Coherent ranges + free dispersal
B Null Model 2: Coherent ranges + bounded dispersal
C Null Model 3: Patchy ranges + free dispersal
D Null Model 4: Patchy ranges + bounded dispersal
Comparative analyses of topographically bounded dispersal constraining species' range shape
Comparative analyses of species range patchiness

fig. S2: Schematic representation of two approaches to simulate species ranges under bounded dispersal. The two panels show the results of a single model iteration based on the empirical distribution of the hummingbird *Metallura phoebe*, found in the Peruvian Andes. Both models simulate ranges starting from a single grid cell (the black grid cell) randomly selected from within the species' empirical range (dotted polygons in 0.25-degree resolution). A: Bounded dispersal as simulated in the main paper (i.e. the geographic dispersal domain, grey zone, includes only the grid cells with elevations that fall within the elevational range limits). B: The geographic dispersal domain additionally excludes grid cells that fall outside the range of climate conditions found within the species' range. The contemporary climate conditions comprise the mean annual temperature, mean annual precipitation, temperature seasonality, and precipitation seasonality. The results show that bounded dispersal according to the species' elevational range limits and contemporary climate volume may both underpredict the empirical range linearity.

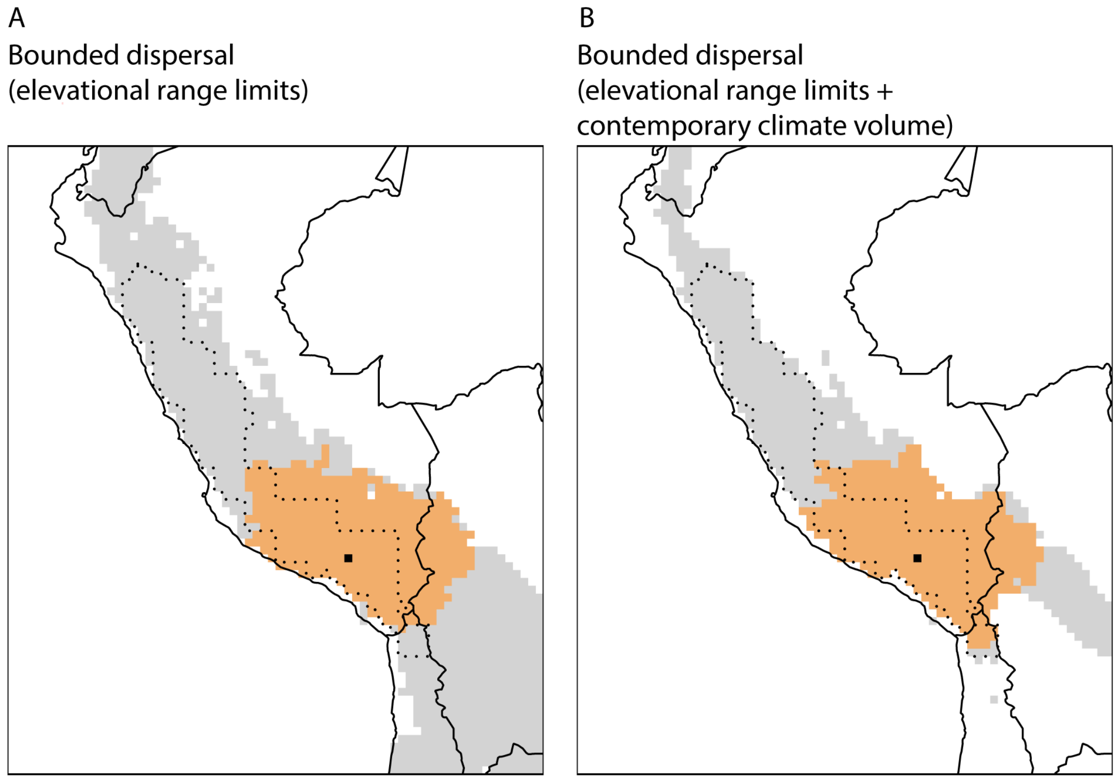

fig. S3: Comparison between simulations using the species' elevational range limits and occupied climate volumes as constraints to bounded dispersal. The scatterplots show the relationship between the total simulated and empirical species richness at a 1-degree spatial resolution. All three simulation models assume multiple geographic starting positions (corresponding to model 4 in fig. S1). The most constrained model, which incorporates both elevational range limits and climate volume, is used for bounded dispersal in the main manuscript.

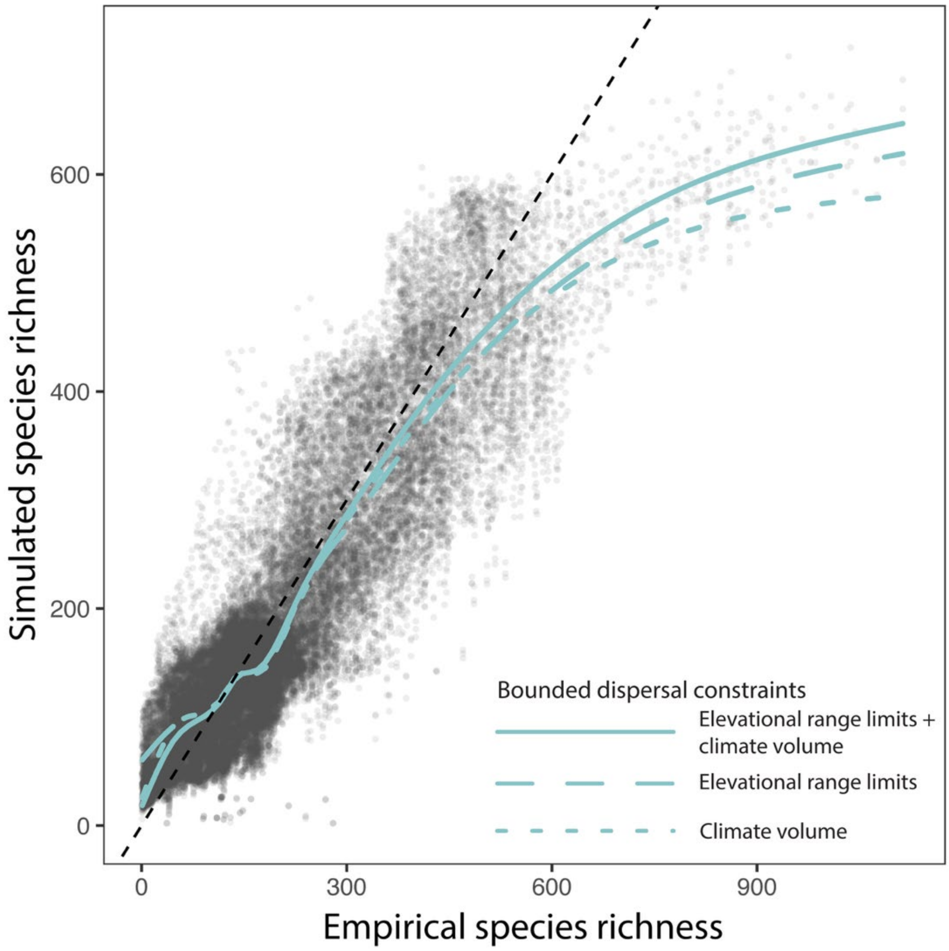

fig. S4: Two-way comparisons of bounded versus free dispersal and coherent versus patchy range shapes.

The difference in predicted species richness between the simulation models (sm) with bounded *vs*. free dispersal (A-B) identifies areas where topographic constraints on range linearity have the greatest influence on species richness. Similarly, the difference in species richness between models with patchy *vs*. coherent ranges (C-D) identifies areas where range patchiness influences species richness. Red colours indicate that the predicted species richness increases, due to range linearity or patchiness, whereas blue colours mean that the species richness decreases. The dark grey shades highlight insular regions that were excluded from the analyses

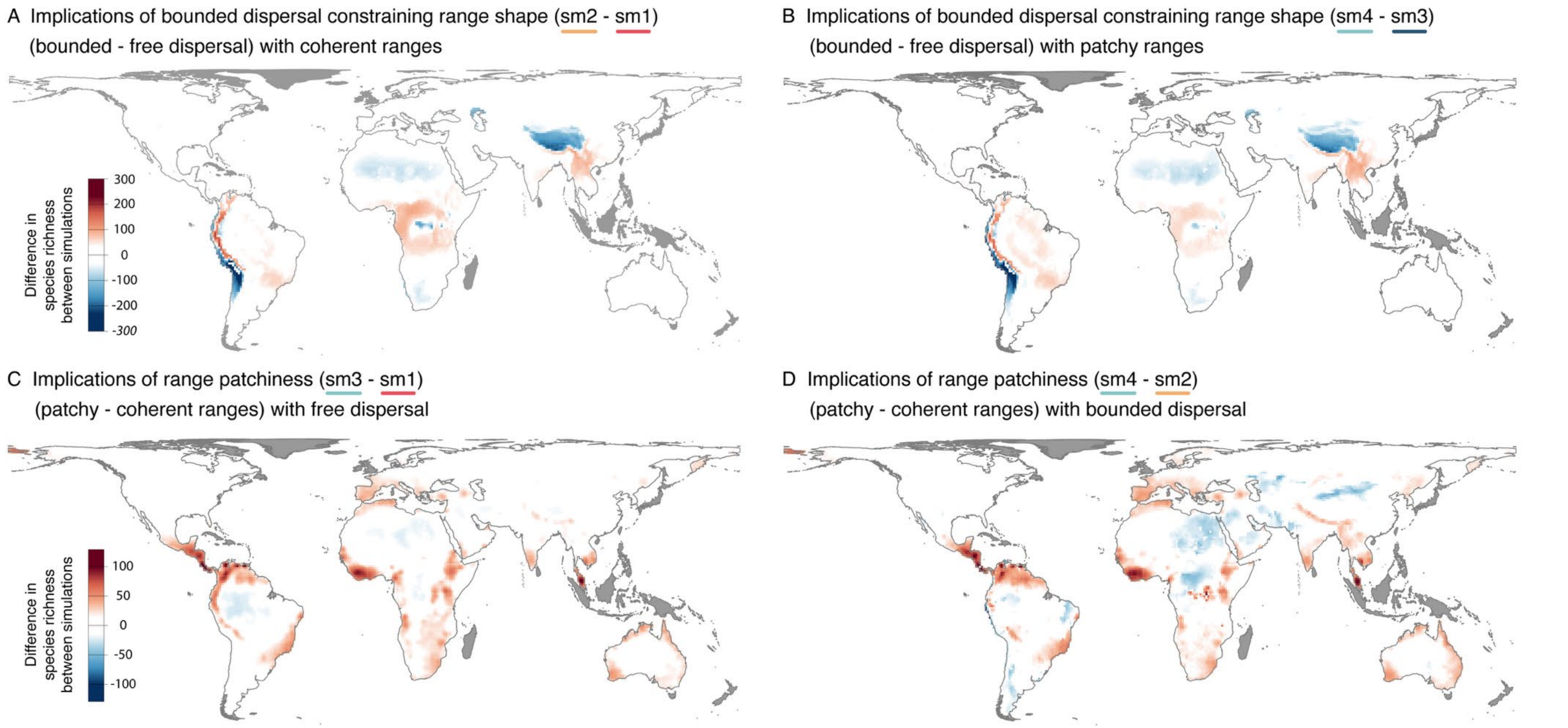

A Implications of bounded dispersal constraining range shape (sm2 - sm1)
(bounded - free dispersal) with coherent ranges
B Implications of bounded dispersal constraining range shape (sm4 - sm3)
(bounded - free dispersal) with patchy ranges
Difference in species richness between simulations
300
200
100
0
-100
-200
-300
C Implications of range patchiness (sm3 - sm1)
(patchy - coherent ranges) with free dispersal
D Implications of range patchiness (sm4 - sm2)
(patchy - coherent ranges) with bounded dispersal
Difference in species richness between simulations
100
50
0
-50
-100

fig. S5: Identifying and aggregating tiny range patches. Examples of species selected from the Northern Andes with tiny range patches that, in our biogeographical simulations, were considered aggregated with a nearby larger range patch (thereby not considered independent range patches in models 3 and 4). Colours on the maps show the species' distinct range patches before and after the aggregation procedure. Black circles highlight tiny patches that were aggregated with nearby patches. See the methods section for a description of the algorithm used to identify and aggregate range patches.

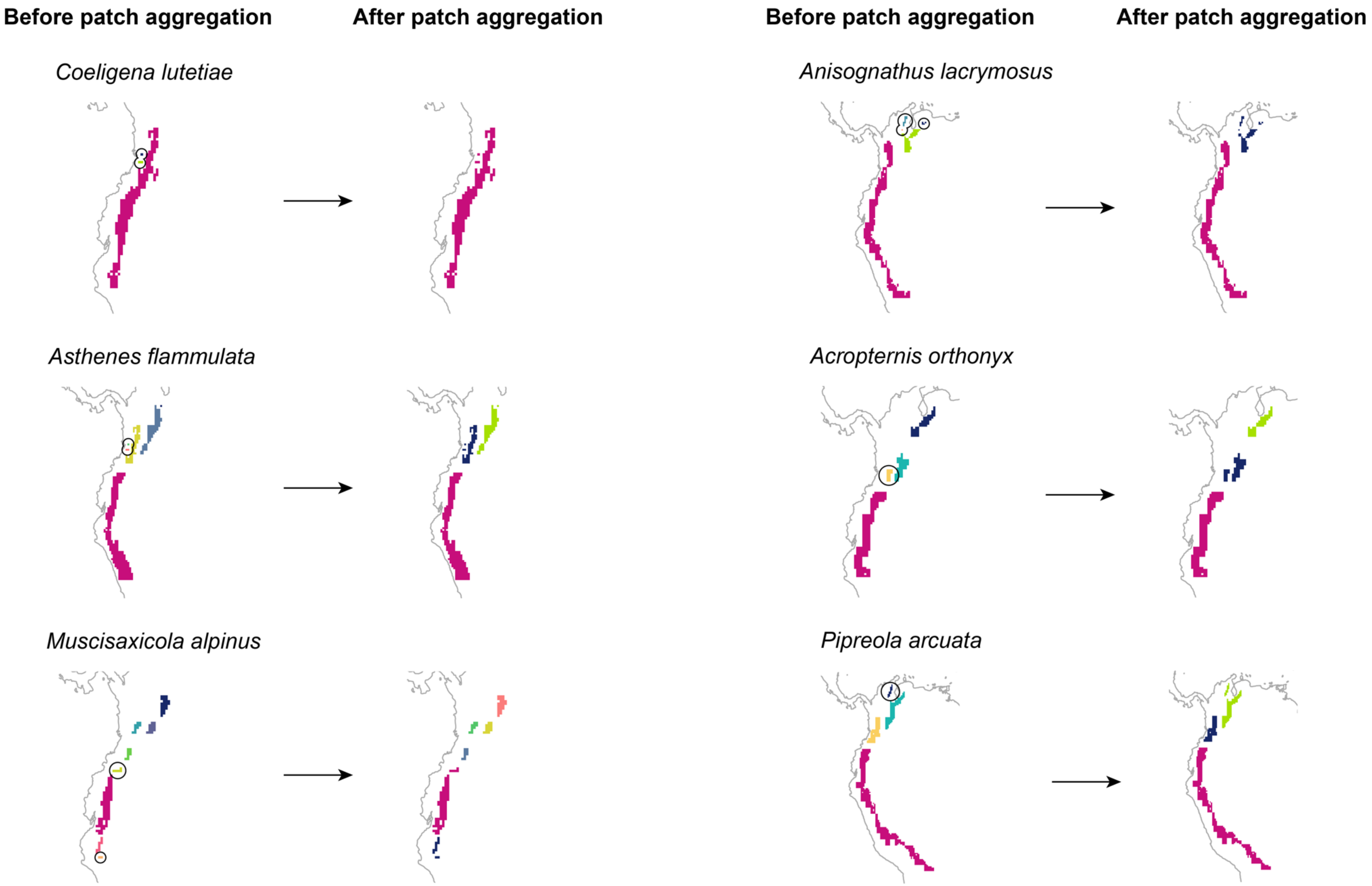
Before patch aggregation
After patch aggregation
Before patch aggregation
After patch aggregation
Coeligena lutetiae
Anisognathus lacrymosus
Asthenes flammulata
Acropternis orthonyx
Muscisaxicola alpinus
Pipreola arcuata

fig. S6: Example of range size standardisation for a focal geographic realm (*i.e.* Palearctic region in Fig. 4). Empirical range sizes (green curve) are matched, in rank order, with 1000 random samples from the global range size distribution. The grey ribbon marks the 95% variation of the samples, and the black line represents the sample mean. Circles mark the empirical and standardised range size for a focal species.

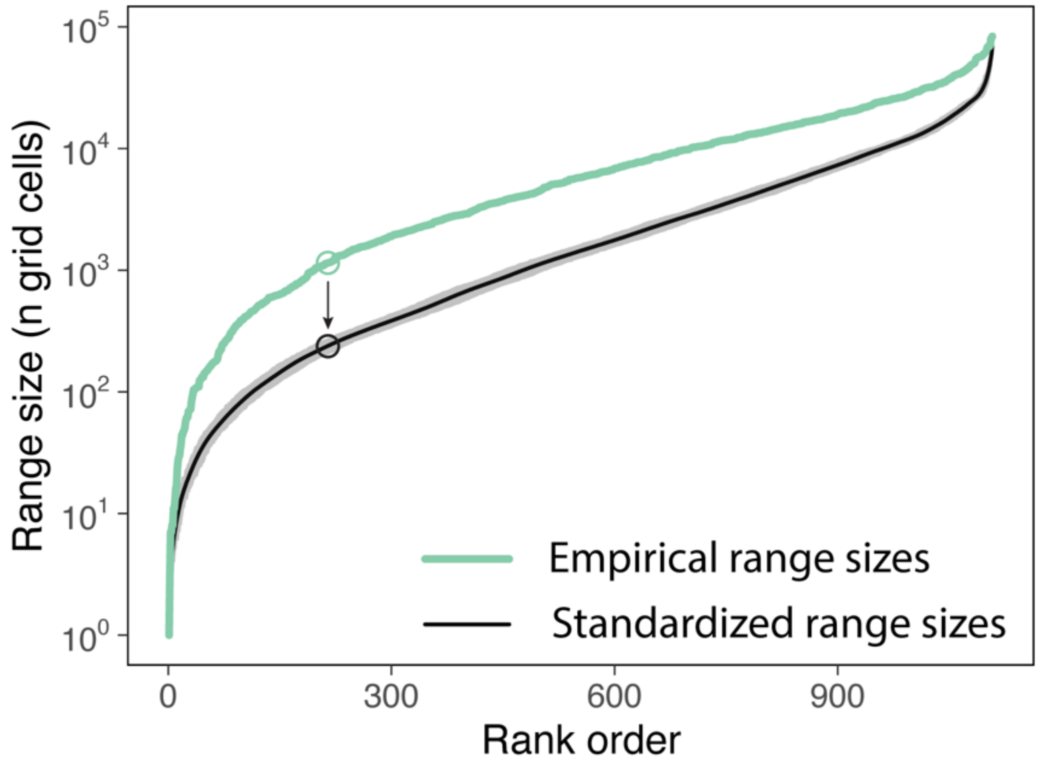

REFERENCES AND NOTES

1. C. Rahbek, M. K. Borregaard, R. K. Colwell, B. Dalsgaard, B. G. Holt, N. Morueta-Holme, D. Nogues-Bravo, R. J. Whittaker, J. Fjeldså, Humboldt's enigma: What causes global patterns of mountain biodiversity? *Science* **365**, 1108–1113 (2019).

2. J. V. Remsen Jr., High incidence of "leapfrog" pattern of geographic variation in Andean birds: Implications for the speciation process. *Science* **224**, 171–173 (1984).

3. G. R. Graves, Linearity of geographic range and Its possible effect on the population structure of Andean birds. *Auk* **105**, 47–52 (1988).

4. J. Fjeldså, N. Krabbe, *Birds of the High Andes* (Zoological Museum, University of Copenhagen and Apollo Books, 1990).

5. J. H. Brown, G. C. Stevens, D. M. Kaufman, The geographic range: Size, shape, boundaries, and internal structure. *Annu. Rev. Ecol. Evol. Syst.* **27**, 597–623 (1996).

6. A. L. Pigot, I. P. F. Owens, C. D. L. Orme, The environmental limits to geographic range expansion in birds. *Ecol. Lett.* **13**, 705–715 (2010).

7. J. H. Brown, B. A. Maurer, Macroecology: The division of food and space among species on continents. *Science* **243**, 1145–1150 (1989).

8. K. Böhning-Gaese, T. Caprano, K. van Ewijk, M. Veith, Range size: Disentangling current Traits and phylogenetic and biogeographic factors. *Am. Nat.* **167**, 555–567 (2006).

9. A. Ruggiero, Size and shape of the geographical ranges of Andean passerine birds: Spatial patterns in environmental resistance and anisotropy. *J. Biogeogr.* **28**, 1281–1294 (2001).

10. E. H. Rapoport, *Areography: Geographical Strategies of Species* (Pergamon Press, 1982).

11. D. H. Janzen, Why mountain passes are higher in the tropics. *Am. Nat.* **101**, 233–249 (1967).

12. G. G. Simpson, Species density of North American recent mammals. *Syst. Zool.* **13**, 57–73 (1964).

13. E. R. Pianka, Latitudinal gradients in species diversity: A review of concepts. *Am. Nat.* **100**, 33–46 (1966).

14. G. C. Stevens, The latitudinal gradient in geographical range: How so many species coexist in the tropics. *Am. Nat.* **133**, 240–256 (1989).

15. R. K. Colwell, G. C. Hurtt, Nonbiological Gradients in Species Richness and a Spurious Rapoport Effect. *Am. Nat.* **144**, 570–595 (1994).

16. C. Rahbek, G. R. Graves, Multiscale assessment of patterns of avian species richness. *Proc. Natl. Acad. Sci. U.S.A.* **98**, 4534–4539 (2001).

17. W. Jetz, C. Rahbek, Geographic Range Size and Determinants of Avian Species Richness. *Science* **297**, 1548–1551 (2002).

18. D. Storch, P. Keil, W. Jetz, Universal species–area and endemics–area relationships at continental scales. *Nature* **488**, 78–81 (2012).

19. G. R. Graves, C. Rahbek, Source pool geometry and the assembly of continental avifaunas. *Proc. Natl. Acad. Sci. U.S.A.* **102**, 7871–7876 (2005).

20. A. Baselga, J. M. Lobo, J.-C. Svenning, M. B. Araújo, Global patterns in the shape of species geographical ranges reveal range determinants. *J. Biogeogr.* **39**, 760–771 (2012).

21. C. D. Cadena, L. N. Céspedes, "Origin of elevational replacements in a clade of nearly flightless birds: Most diversity in tropical mountains accumulates via secondary contact following allopatric speciation" in *Neotropical Diversification: Patterns and Processes* (Springer, 2020), pp. 635–659.

22. J. Sonne, C. Rahbek, Idiosyncratic patterns of local species richness and turnover define global biodiversity hotspots. *Proc. Natl. Acad. Sci. U.S.A.* **121**, e2313106121 (2024).

23. I. R. McFadden, B. Sandel, C. Tsirogiannis, N. Morueta-Holme, J.-C. Svenning, B. J. Enquist, N. J. B. Kraft, Temperature shapes opposing latitudinal gradients of plant taxonomic and phylogenetic β diversity. *Ecol. Lett.* **22**, 1126–1135 (2019).

24. C. Lauzeral, G. Grenouillet, S. Brosse, Spatial range shape drives the grain size effects in species distribution models. *Ecography* **36**, 778–787 (2013).

25. C. Rahbek, L. Hansen, J. Fjeldså, *One Degree Resolution Database of the Global Distribution of Birds* (Natural History Museum of Denmark, University of Copenhagen, 2012).

26. B. G. Holt, J.-P. Lessard, M. K. Borregaard, S. A. Fritz, M. B. Araújo, D. Dimitrov, P.-H. Fabre, C. H. Graham, G. R. Graves, K. A. Jønsson, D. Nogués-Bravo, Z. Wang, R. J. Whittaker, J. Fjeldså, C. Rahbek, An Update of Wallace's Zoogeographic Regions of the World. *Science* **339**, 74–78 (2013).

27. J. Sonne, B. Dalsgaard, M. K. Borregaard, J. Kennedy, J. Fjeldså, C. Rahbek, Biodiversity cradles and museums segregating within hotspots of endemism. *Proc. Biol. Sci.* **289**, 20221102 (2022).

28. B. H. Warren, D. Simberloff, R. E. Ricklefs, R. Aguilée, F. L. Condamine, D. Gravel, H. Morlon, N. Mouquet, J. Rosindell, J. Casquet, E. Conti, J. Cornuault, J. M. Fernández-Palacios, T. Hengl, S. J. Norder, K. F. Rijsdijk, I. Sanmartín, D. Strasberg, K. A. Triantis, L. M. Valente, R. J. Whittaker, R. G. Gillespie, B. C. Emerson, C. Thébaud, Islands as model systems in ecology and evolution: Prospects fifty years after MacArthur-Wilson. *Ecol. Lett.* **18**, 200–217 (2015).

29. A. Castro-Insua, C. Gómez-Rodríguez, J.-C. Svenning, A. Baselga, A new macroecological pattern: The latitudinal gradient in species range shape. *Glob. Ecol. Biogeogr.* **27**, 357–367 (2018).

30. W. Jetz, C. Rahbek, Geometric constraints explain much of the species richness pattern in African birds. *Proc. Natl. Acad. Sci. U.S.A.* **98**, 5661–5666 (2001).

31. W. E. Kunin, Sample shape, spatial scale and species counts: Implications for reserve design. *Biol. Conserv.* **82**, 369–377 (1997).

32. A. H. Hurlbert, W. Jetz, Species richness, hotspots, and the scale dependence of range maps in ecology and conservation. *Proc. Natl. Acad. Sci. U.S.A.* **104**, 13384–13389 (2007).

33. M. T. P. Coelho, E. Barreto, T. F. Rangel, J. A. F. Diniz-Filho, R. O. Wüest, W. Bach, A. Skeels, I. R. McFadden, D. W. Roberts, L. Pellissier, N. E. Zimmermann, C. H. Graham, The geography of climate and the global patterns of species diversity. *Nature* **622**, 537–544 (2023).

34. R. K. Colwell, Spatial scale and the synchrony of ecological disruption. *Nature* **599**, E8–E10 (2021).

35. B. J. McGill, Ecology. Matters of scale. *Science* **328**, 575–576 (2010).

36. C. D. Cadena, K. H. Kozak, J. P. Gómez, J. L. Parra, C. M. McCain, R. C. K. Bowie, A. C. Carnaval, C. Moritz, C. Rahbek, T. E. Roberts, N. J. Sanders, C. J. Schneider, J. VanDerWal, K. R. Zamudio, C. H. Graham, Latitude, elevational climatic zonation and speciation in New World vertebrates. *Proc. Biol. Sci.* **279**, 194–201 (2012).

37. K. H. Kozak, J. J. Wiens, Niche Conservatism Drives Elevational Diversity Patterns in Appalachian Salamanders. *Am. Nat.* **176**, 40–54 (2010).

38. M. K. Borregaard, G. R. Graves, C. Rahbek, Dispersion fields reveal the compositional structure of South American vertebrate assemblages. *Nat. Commun.* **11**, 491 (2020).

39. A. L. Pigot, J. A. Tobias, Species interactions constrain geographic range expansion over evolutionary time. *Ecol. Lett.* **16**, 330–338 (2013).

40. J. Grinnell, Field tests of theories concerning distributional control. *Am. Nat.* **51**, 115–128 (1917).

41. C. Hoorn, A. Perrigo, A. Antonelli, *Mountains, Climate and Biodiversity* (John Wiley & Sons, 2018).

42. S. G. A. Flantua, A. O'Dea, R. E. Onstein, C. Giraldo, H. Hooghiemstra, The flickering connectivity system of the north Andean páramos. *J. Biogeogr.* **46**, 1808–1825 (2019).

43. C. D. L. Orme, R. G. Davies, M. Burgess, F. Eigenbrod, N. Pickup, V. A. Olson, A. J. Webster, T.-S. Ding, P. C. Rasmussen, R. S. Ridgely, A. J. Stattersfield, P. M. Bennett, T. M. Blackburn, K. J. Gaston, I. P. F. Owens, Global hotspots of species richness are not congruent with endemism or threat. *Nature* **436**, 1016–1019 (2005).

44. F. Gill, D. Dorsker, IOC World Bird Lost (v.10.2) (2020).

45. C. Rahbek, J. Fjeldså, P. A. Hosner, J. Sonne, L. Hansen, "Global database on the elevational distribution of all the bird species of the world," Center for Macroecology, University of Copenhagen, Denmark (2023).

46. National Geophysical Data Center, *2-Minute Gridded Global Relief Data (ETOPO2) v2* (National Geophysical Data Center, NOAA, 2006).

47. C. Rahbek, The role of spatial scale and the perception of large-scale species-richness patterns. *Ecol. Lett.* **8**, 224–239 (2005).

48. M. L. Rosenzweig, *Species Diversity in Space and Time* (Cambridge Univ. Press, 1995).

49. M. K. Borregaard. 2023 "SpreadingDye.jl," February 2024; https://github.com/mkborregaard/SpreadingDye.jl.

50. P. Brun, N. E. Zimmermann, C. Hari, L. Pellissier, D. N. Karger, CHELSA-BIOCLIM+ A novel set of global climate-related predictors at kilometre-resolution (2022).

51. D. Bates, M. Mächler, B. M. Bolker, S. C. Walker, Fitting linear mixed-effects models using lme4. *J. Stat. Softw.* **67**, (2014).

52. R. V. Lenth. "emmeans: Estimated marginal means, aka least-squares means" R package version 1.11.1 (2025).

53. D. F. Stotz, J. W. Fitzpatrick, T. A. Parker III, D. K. Moskovits, *Neotropical Birds: Ecology and Conservation* (University of Chicago Press, 1996).
